# The forsaken mental health of the Indigenous Peoples - a moral case of outrageous exclusion in Latin America

**DOI:** 10.1186/1472-698X-9-27

**Published:** 2009-10-29

**Authors:** Mario Incayawar, Sioui Maldonado-Bouchard

**Affiliations:** 1 Runajambi - Institute for the Study of Quichua Culture and Health, Otavalo, Ecuador

## Abstract

**Background:**

Mental health is neglected in most parts of the world. For the Indigenous Peoples of Latin America, the plight is even more severe as there are no specific mental health services designed for them altogether. Given the high importance of mental health for general health, the status quo is unacceptable. Lack of research on the subject of Indigenous Peoples' mental health means that statistics are virtually unavailable. To illustrate their mental health status, one can nonetheless point to the high rates of poverty and extreme poverty in their communities, overcrowded housing, illiteracy, and lack of basic sanitary services such as water, electricity and sewage. At the dawn of the XXI century, they remain poor, powerless, and voiceless. They remain severely excluded from mainstream society despite being the first inhabitants of this continent and being an estimated of 48 million people. This paper comments, specifically, on the limited impact of the Pan American Health Organization's mental health initiative on the Indigenous Peoples of Latin America.

**Discussion:**

The Pan American Health Organization's sponsored workshop "Programas y Servicios de Salud Mental en Communidades Indígenas" [Mental Health Programs and Services for the Indigenous Communities] in the city of Santa Cruz, Bolivia on July16 - 18, 1998, appeared promising. However, eleven years later, no specific mental health program has been designed nor developed for the Indigenous Peoples in Latin America. This paper makes four specific recommendations for improvements in the approach of the Pan American Health Organization: (1) focus activities on what can be done; (2) build partnerships with the Indigenous Peoples; (3) consider traditional healers as essential partners in any mental health effort; and (4) conduct basic research on the mental health status of the Indigenous Peoples prior to the programming of any mental health service.

**Summary:**

The persistent neglect of the Indigenous Peoples' mental health in Latin America raises serious concerns of moral and human rights violations. Since the Pan American Health Organization' Health of the Indigenous Peoples Initiative 16 years ago, no mental health service designed for them has yet been created.

## Background

According to the World Health Report 2001 of the World Health Organization, at present, 450 million people suffer from a mental or behavioral illness worldwide. Yet most countries allocate less than one percent in budget expenditure for mental health. Advances in biological and behavioral sciences have underscored the firm relationship between social, physical and psychological well-being, further confirming that the current state of neglect of mental health is unacceptable. The situation is even more alarming for the Indigenous Peoples of Latin America, for whom there are no specifically designed mental health programs at all.

Today, the Indigenous Peoples of Latin America, estimated at around 48 millions, remain poor, powerless, and excluded from a society which consistently neglects their healthcare needs[1,2]. During nearly 200 years as independent states, the dominant, non-indigenous society has governed the countries of Latin America, and has held little to no regard for the well-being and mental health of the Indigenous Peoples. The Indigenous Peoples' plight remains more often than not invisible, for the dominant Latin American society often considers them as almost extinct by the force of historical events. Moreover, outside observers often mistake them for an integral part of the dominant, non-indigenous population of Latin America, though there have recently been some extraordinary cases of Indigenous Peoples gaining a voice, such as is the case in Bolivia for President Evo Morales.

As recently stated by Health Unlimited (a British non-governmental organization) and the London School of Hygiene and Tropical Medicine, Indigenous Peoples are unable to access routine health care; they are dying prematurely, despite efforts by the United Nations, the Pan American Health Organization (PAHO), and their member states[3]. Because there are too few studies on the subject, there are hardly any statistics on the mental health of the Indigenous Peoples[4,5]. A recent review underscored the scarcity of studies dealing with the mental health of Indigenous Peoples in Latin America and their access to psychiatric services[5]. It is known, however, that for the estimated five million Indigenous people in Ecuador, there are only five Quichua physicians, of which only one is a psychiatrist [6]. Some statistics on general health further illustrate the predicament. For example, the infant mortality rate for Quichuas in northern Ecuador was 211 per 1000 in 1986, while it was 38 per 1000 for the country as a whole[5]. The Mission Report for the World Food Program in Guatemala indicated that 95 percent of Indigenous Peoples of 14 years or less suffer from malnutrition[5]. In Venezuela, researchers reported in 2002 a rate of alcoholism as high as 86.5% for men in an Indigenous community[7]. In Mexico, almost 90% of indigenous communities live in extreme poverty[8]. According to the World Bank, geographic regions of poverty in Latin America are precisely those in which Indigenous communities live, and the use of an indigenous language in a household closely associates with poverty[9,10]. Indeed, the researchers who do investigate the socioeconomic situation of Indigenous Peoples of Latin America have come to paint what is a sad portrait of inequity for these countries. Available data on Indigenous communities invariably show high rates of poverty and extreme poverty, overcrowded housing, illiteracy, and lack of basic sanitary services such as water, electricity and sewage. These extreme inequitable social, health and economic conditions are at the heart of the social exclusion experienced by the Indigenous Peoples[11].

It is worth noting that the Article 24.2 of the United Nations Declaration on the Rights of Indigenous Peoples adopted in 2007 stipulates that "Indigenous individuals have an equal right to the enjoyment of the highest attainable standard of physical and mental health. States shall take the necessary steps with a view to achieving progressively the full realization of this right." It is the case that the neglect of Indigenous Peoples' mental health poses not only a social and health problem, but critically, raises a basic human rights and ethics issue[12].

## Discussion

### The Latin American countries promise

PAHO made an unexpected move eleven years ago with its unique mental health services meeting in Bolivia. It sponsored a workshop called "Programas y Servicios de Salud Mental en Communidades Indígenas" [Mental Health Programs and Services for the Indigenous Communities] in the city of Santa Cruz, Bolivia on July 16 - 18, 1998 [13]. The workshop was part of PAHO's Health of Indigenous People Initiative, created in September 1993. It was also a response to the emerging global interest in solving the human rights, environmental, educational and health problems faced by the Indigenous Peoples, as the United Nations had stated in 1995, launch year of the international Decade of the World's Indigenous Peoples (1995-2004).

PAHO's goal and that of the Bolivian government, who hosted the meeting, was to lay the ground for the implementation of mental health programs and services for the Indigenous Peoples of the region. For the first time, some Indigenous Peoples were present and partook in the small workshop (30 participants). Countries present included Bolivia, Brazil, Chile, Ecuador, the Unites States of America, Guatemala, Mexico, Nicaragua, and Peru, as well as nine PAHO officials.

Enthusiastically, PAHO defined the following objectives:

▪ Creation of a flexible mental health system adapted to each country and directed to Indigenous Peoples.

▪ Acknowledgement of the importance of traditional medicine to provide mental health services and training.

▪ Cooperation between official Western medicine and traditional medicine.

▪ Improvement of access to the existing services in order to solve the current unacceptable health disparities between Indigenous communities and the rest of the population.

▪ Obligation for the member States to promote intercultural understanding and respect of Indigenous Peoples' culture.

▪ Decentralization and regionalization of mental health services.

▪ Integration of mental health services in the existing primary care services.

▪ Community participation in the design, administration and evaluation of mental health services.

▪ Training of mental health workers.

▪ Implementation of mental health programs with the participation of Indigenous Peoples' organizations.

▪ Provision of culture-sensitive services for Indigenous Peoples.

▪ Modification of university curricula to incorporate the notions of interculturalism, social factors in health and disease, and traditional medicine.

▪ Integration of the mental health field in PAHO's training of health professionals.

▪ Provision of technical assistance and follow-up by PAHO for national and international efforts in mental health.

▪ Organization by PAHO of a meeting of traditional healers.

▪ Creation by PAHO of a coordinating group PAHO/Indigenous communities and a Council of Traditional medicine of the Americas.

### Broken promises?

Recently, PAHO reported a list of progressive actions in its Health of Indigenous People Initiative: Strategic Directions and Plan of Actions 2003-2007[14]. However, eleven years after the Workshop on Mental Health Services held in Bolivia, specific mental health programs designed for Indigenous Peoples have yet to be created in Latin America.

Looking at the outcomes of the 16 years old PAHO initiative, the ten million dollar budget allocated for the entire Health of the Indigenous People Initiative seems to have been spent mainly on technical reports, meetings, activity planning, and other bureaucratic tasks. Although PAHO has created the Health of the Indigenous Peoples Unit, legitimate participation of Indigenous Peoples is lacking. Interestingly, in the last couple of years, several countries have created specific units that are meant to deal with Indigenous Peoples' health in general, with no regards to mental health. In Ecuador, for example, the national Intercultural Health program, part of the Ministry of Health, was given the mission of "defining public policies to protect, recover, and develop the traditional medicine of the country." Unfortunately, political partisanship soon undermined its operation as it became the main criteria for staff selection and recruitment, rather than health and mental health expertise.

Organizations such as PAHO, ministries of health, and mental health care services remain hermetic and distant to the Indigenous Peoples. Moreover, local governments often consider Indigenous Peoples as second class citizens, outsiders, and inferiors[4]. Consequently, they are literally excluded from the conventional Western health services. At the local level, many Indigenous patients still perceive hospitals, clinics, and primary care health posts as dangerous places where they undergo the risk of being inadequately treated, humiliated, insulted or even killed by racist doctors[5,15]. They are scared to visit hospitals or doctors. As observed by the senior author (MI), they sometimes even refuse to seek medical care, preferring to die at home. The Indigenous Peoples have learned, through a history of social exclusion, racial discrimination, dispossession, and violence, to be suspicious of the dominant society's intentions in their regard. They view the Latin American governments as entities that do not represent their interests, but rather that perpetuate a policy of centuries-long colonial oppression[5]. The Indigenous Peoples' views and health seeking behavior is probably the result of these historical experiences.

### What could be done?

PAHO's initial efforts could yield some positive mental health outcomes, granted it is willing to:

▪ Avoid overenthusiastic statements on what could be done, and focus on what can be achieved.

▪ Build active partnership with the intended target population. It is inappropriate to work exclusively with government health bureaucrats (often members of the dominant Spanish-speaking mainstream society). Doing so leads to Indigenous Peoples viewing PAHO's actions as patronizing, and its program as governed by outsiders. It is imperative to work with the Indigenous researchers, physicians, mental health professionals, and other Indigenous communities' representatives.

▪ Consider traditional healers as essential partners in any mental health effort, including delivering health services, implementing psychiatric research, and formulating mental health policies[6].

▪ Fund a critical number of basic research studies on the mental health status of the Indigenous Peoples prior to any mental health service programming. Delegate those studies to Indigenous health research centers. This would prevent doing what PAHO has done in the past 10 years; that is, proposing solutions before having a basic knowledge of the prevalence of mental illness, the local notions of well-being, the local experience of illness, and the mental health needs of the Indigenous Peoples. Commendable efforts are currently being made in Canada and the USA to tackle these mental health problems through research. The Institute of Aboriginal Peoples' Health, part of the Canadian Institutes of Health Research and the Native American Research Center for Health initiative, a partnership of the Indian Health Service and the National Institutes of Health, are government research funding models that could be followed by the Latin American States.

In the present Latin American social climate, with countries literally excluding Indigenous Peoples from society, PAHO's promises seem too good to be true. Change in the basic ways PAHO operates with the Indigenous communities is vital. The disconnection between PAHO and its target population ultimately results in the failure of its efforts and the waste of scarce resources. To prevent this, Indigenous Peoples should be included in any program suggested. In New Zealand, for example, the Ministry of Health has funded a two million dollars per year initiative to allow native Maori healers to provide healing services alongside conventional Western medicine[16]. At the dawn of the XXI century, the pervasive neglect of Indigenous Peoples' mental health raises concerns of human rights violations and illustrates the failure of Latin American countries to address the Indigenous Peoples' basic right to receive health and mental health services. The moral case is to be made[12].

The Indigenous Peoples, the founding inhabitants of this continent, deserve some relief from the centuries-long colonization, oppression, and land dispossession. The current Latin American states' moral numbness to the mental health needs of Indigenous Peoples must be highlighted. The Indigenous Peoples continue, as they did for centuries or millennia, to depend entirely on traditional healers, family, and community support to cope with their mental health problems and to relieve their psychological distress.

## Summary

1. Latin American countries completely neglect the mental health of the 48 million Indigenous Peoples.

2. Despite PAHO's 16 years old Health of the Indigenous Peoples Initiative, today, there are still no mental health services designed for Indigenous Peoples.

3. It is recommended that PAHO improve its direct partnership with the Indigenous Peoples, including traditional healers, Indigenous mental health professionals and community representatives.

4. The persistent neglect of Indigenous Peoples' mental health raises serious concerns of moral and human rights violations.

## Competing interests

The authors declare that they have no competing interests. Dr. Mario Incayawar founded the Jambihuasi integrative (Quichua-Western) health center in Otavalo, Ecuador 25 years ago, but holds no role in the center today.

## Authors' contributions

MI formulated the original idea for this debate article, and wrote the initial draft, and SMB critically reviewed the manuscript and contributed additional ideas. All authors read and approved the final manuscript.

## Authors' informations

Mario Incayawar, M.D., M.Sc., DESS., is the Director of the Runajambi Institute for the Study of Quichua Culture and Health, Otavalo, Ecuador. He is the former Henry R. Luce Professor in Brain, Mind and Medicine: Cross-Cultural Perspectives at Pitzer, Claremont McKenna, and Harvey Mudd Colleges, Claremont, California, USA. http://www.runajambi.org/incayawar/

Sioui Maldonado Bouchard is a Research Assistant at the Runajambi Institute for the Study of Quichua Culture and Health. She is completing her Masters' degree in psychology at the Université de Montréal, Montreal, Canada.

## Pre-publication history

The pre-publication history for this paper can be accessed here:

http://www.biomedcentral.com/1472-698X/9/27/prepub
